# Cardiac steatosis and left ventricular function in men with metabolic syndrome

**DOI:** 10.1186/1532-429X-15-103

**Published:** 2013-11-14

**Authors:** Kristofer Nyman, Marit Granér, Markku O Pentikäinen, Jesper Lundbom, Antti Hakkarainen, Reijo Sirén, Markku S Nieminen, Marja-Riitta Taskinen, Nina Lundbom, Kirsi Lauerma

**Affiliations:** 1 Department of Radiology, HUS Medical Imaging Center, Helsinki University Central Hospital and University of Helsinki, Stenbäckinkatu 11, BOX 281, Helsinki FI-00029 HUS, Finland; 2 Heart and Lung Center, Division of Cardiology, Helsinki University Central Hospital and University of Helsinki, Helsinki, Finland; 3 Department of General Practice and Primary Health Care, Health Care Centre of City of Helsinki and University of Helsinki, Helsinki, Finland; 4 Department of Radiology, HUS Medical Imaging Center, Helsinki University Central Hospital and University of Helsinki, Haartmaninkatu 4, BOX 340, FI-00029 HUS, Finland

**Keywords:** Cardiovascular magnetic resonance, Proton magnetic resonance spectroscopy, Metabolic syndrome, Obesity, Diastolic dysfunction, Myocardial triglyceride content, Epicardial fat, Pericardial fat, Cardiac steatosis

## Abstract

**Background:**

Ectopic accumulation of fat accompanies visceral obesity with detrimental effects. Lipid oversupply to cardiomyocytes leads to cardiac steatosis, and in animal studies lipotoxicity has been associated with impaired left ventricular (LV) function. In humans, studies have yielded inconclusive results. The aim of the study was to evaluate the role of epicardial, pericardial and myocardial fat depots on LV structure and function in male subjects with metabolic syndrome (MetS).

**Methods:**

A study population of 37 men with MetS and 38 men without MetS underwent cardiovascular magnetic resonance and proton magnetic spectroscopy at 1.5 T to assess LV function, epicardial and pericardial fat area and myocardial triglyceride (TG) content.

**Results:**

All three fat deposits were greater in the MetS than in the control group (p <0.001). LV diastolic dysfunction was associated with MetS as measured by absolute (471 mL/s vs. 667 mL/s, p = 0.002) and normalized (3.37 s^-1^ vs. 3.75 s^-1^, p = 0.02) LV early diastolic peak filling rate and the ratio of early diastole (68% vs. 78%, p = 0.001). The amount of epicardial and pericardial fat correlated inversely with LV diastolic function. However, myocardial TG content was not independently associated with LV diastolic dysfunction.

**Conclusions:**

In MetS, accumulation of epicardial and pericardial fat is linked to the severity of structural and functional alterations of the heart. The role of increased intramyocardial TG in MetS is more complex and merits further study.

## Background

Cardiovascular diseases are a common co-morbidity of the worldwide obesity epidemic. Abdominal obesity in particular associates with metabolic syndrome (MetS) and type 2 diabetes mellitus (T2DM). Excess calorie intake and sedentary lifestyle combined with unfavorable genotype and several environmental factors result in lipid overflow, due to a failure of subcutaneous adipose tissue to expand and store the excess of circulating free fatty acids (FFA). Consequently, ectopic fat accumulates around the viscera and into sites regularly containing only minor amount of adipose tissue, such as the liver, pancreas, skeletal muscle, and heart [1,2]. Ectopic fat deposits have been subdivided into those with local and those with systemic effects [2,3]. According to this, perivascular, myocardial, and epi/pericardial fat have mainly local unfavorable effects, whereas visceral adipose tissue, or fat in the liver or skeletal muscles have systemic effects due to the fundamental role of these organs in glucose, insulin, and lipid metabolism. In this context, both the amount and location of ectopic adipose tissue are highly important with respect to the cardiovascular morbidity and mortality.

Heart-related fat can be subdivided into myocardial, epicardial, and pericardial fat [4]. In recent years, ^1^H-magnetic resonance spectroscopy (^1^H-MRS) has proved to be a reliable method to noninvasively quantify cardiomyocytic triglyceride (TG) content *in vivo*[5]. Increased cardiac adiposity has been associated with obesity, impaired glucose tolerance, and T2DM [6-8]. Animal studies have provided evidence on a close relationship between cardiac lipotoxicity and impaired left ventricular (LV) function [9]. In T2DM patients, myocardial TG content associates with LV diastolic dysfunction [10,11]. The mechanism behind this phenomenon has remained unresolved, and also controversial reports have been published [8]. To our knowledge, only limited data exist on the relationship of cardiac steatosis and diastolic LV function in non-diabetic male subjects with MetS.

Subclinical LV dysfunction has been reported to associate with obesity and MetS by means of other imaging modalities [12,13]. However, cardiovascular magnetic resonance (CMR) studies allowing precise LV filling pattern analysis are limited [14,15]. The present study focuses on the LV diastolic function with specific interest on the role of all three cardiac fat compartments in male subjects with MetS.

## Methods

### Study population

**Figure 1:**
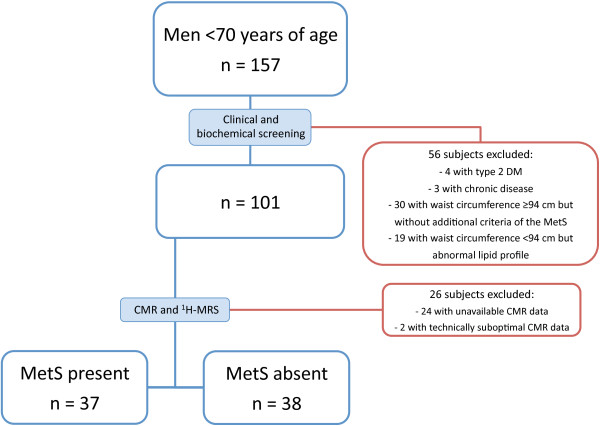
**Details of study design.** Blue boxes indicate the number of study subjects at each stage and boxes outlined in red indicate drop-out rates at each stage.

Male subjects were recruited by advertisements in local newspapers. The study recruitment is summarized and the rates of drop out at each stage are shown in Figure 1. The final study population consisted of 75 Finnish men of Caucasian ethnicity. Based on cardiometabolic risk factors, subjects were divided into two groups: those with MetS and those without MetS. To qualify for the MetS group, subjects must have waist circumference ≥ 94 cm in addition to two or more abnormal findings according to the harmonized definition of MetS [16]. The other subjects were classified as subjects without MetS. Exclusion criteria from the study included the following: other known acute or chronic disease based on medical history, physical examination, and standard laboratory tests (blood counts, creatinine, aspartate aminotransferase, alanine aminotransferase, thyroid-stimulating hormone), T2DM (based on a 2-h oral glucose tolerance test), significant alcohol consumption (more than 20 grams per day), and treatment with other lipid lowering therapy than statins. As the hormonal status and use of contraceptives modify lipid metabolism in women, only male subjects were recruited. Smoking and elevated liver enzymes were allowed. Five study subjects were on regular medication for hypertension, three for dyslipidemia (statins), and one for both hypertension and dyslipidemia. In participants with MetS, coronary artery disease (CAD) was additionally excluded by adenosine stress perfusion CMR followed by late gadolinium enhancement images. The rationale for this was to exclude the potential interfering effects of CAD-associated myocardial scar tissue and/or altered cardiac function or lipid metabolism caused by myocardial ischemia. The study was approved by the Ethics Committee of the Department of Medicine, Hospital District of Helsinki and Uusimaa, and each subject provided written informed consent to participate.

### Demographic variables and biochemical investigations

Body mass index (BMI) was calculated by dividing weight in kilograms by the square of the height in meters (kg/m^2^). Waist circumference was measured at a level midway between the lower rib lateral margin and the iliac crest in the horizontal position. Blood pressure was recorded as an average of five measurements obtained in the sitting position after a 5 min rest using a BPM-200 monitor (Quick Medical, WA, USA). The subjects were classified as present, past, or non-smokers.

Blood samples were collected after an overnight fast. Total serum cholesterol, TGs, and high-density lipoprotein cholesterol were measured by Konelab analyzer 60i with Konelab TM kits (both from Thermo Fisher Scientific, Finland). The concentration of low-density lipoprotein cholesterol was derived from the Friedewald formula [17]. Fasting and postload glucose were assessed by the hexokinase method (Gluco-quant, Roche Diagnostics, Basel, Switzerland) using either a Hitachi 917 or Modular analyzer (both from Hitachi Ltd, Tokyo, Japan). Serum insulin concentration was determined by double-antibody radioimmunoassay (Pharmacia RIA kit, Pharmacia, Uppsala, Sweden). The insulin-resistance homeostasis model assessment (HOMA) index was calculated by using the formula: (fasting plasma glucose x fasting plasma insulin)/22.5 [18].

### CMR protocol

Cardiac imaging was performed with a 1.5 Tesla whole-body MR scanner (Magnetom Avanto; Siemens, Erlangen, Germany) with subject lying at rest in supine position. A multi-channel body coil was used for reception. Cine series were acquired in 4-chamber, 2-chamber and LV short axis orientations during breath hold using a retrospectively electrocardiographically gated steady state free precession gradient echo sequence. A stack of short axis cine series (typically 12 slices) was obtained covering the LV from base to apex with typical imaging parameters of repetition time 50 ms, echo time 1.18 ms, flip angle 69 degrees, matrix 186 × 220, field of view 355 × 420 mm, slice thickness 8 mm, gap 2 mm, and temporal resolution 32–53 ms.

### Image analysis

**Figure 2:**
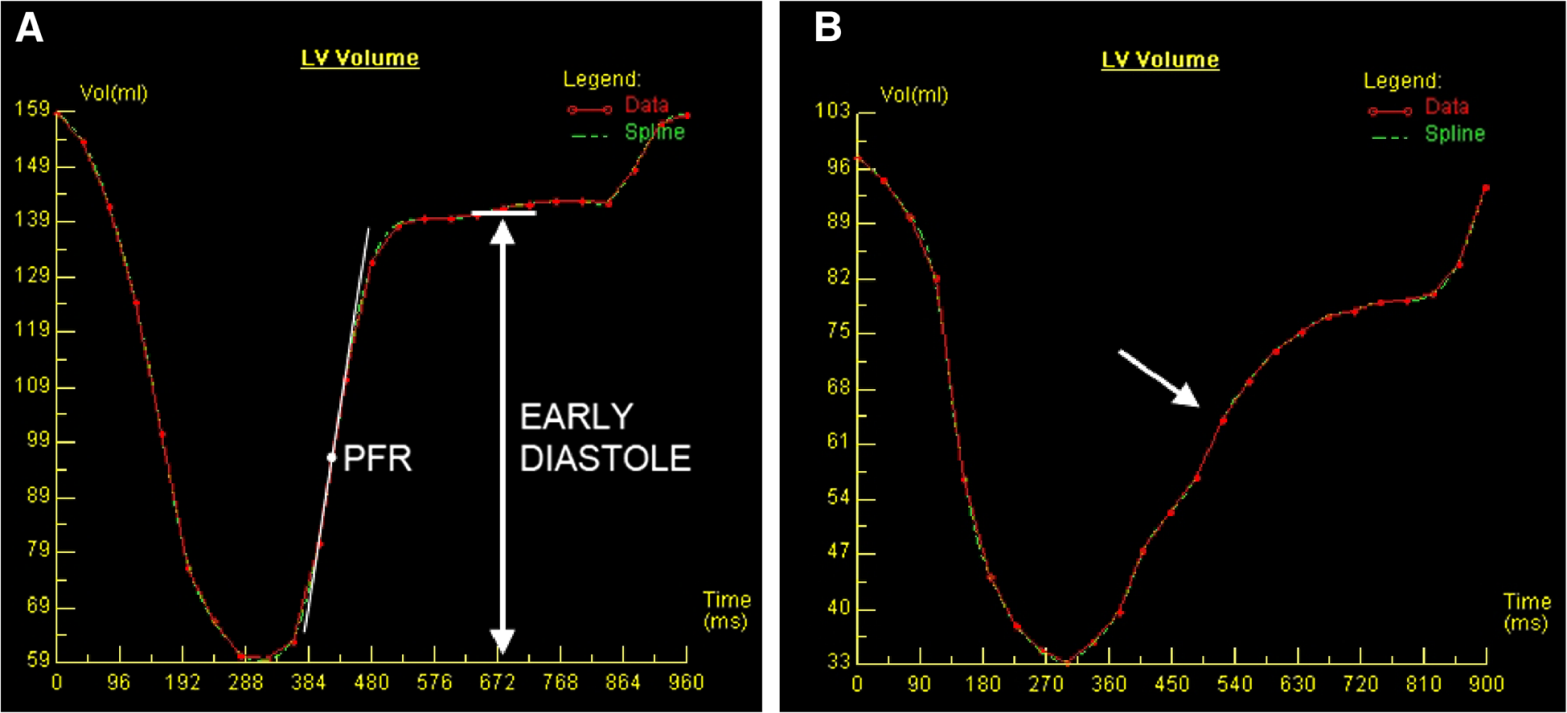
**Evaluation of diastolic function in left ventricular (LV) volume versus time curve. A)** LV filling pattern in a normal subject. Early peak filling rate (PFR) is derived from the steepest gradient in the volume curve in the early filling phase. The horizontal white line demonstrates the diastolic diastasis phase (plateau) separating the early and late diastole. **B)** LV filling pattern in a metabolic syndrome patient with LV diastolic dysfunction demonstrating a depression of diastolic plateau and early PFR (arrow).

Dedicated post-processing software (Argus; Siemens Medical Solutions, Erlangen, Germany) was used to perform a volumetric analysis of the LV. The analysis was performed by two radiologists with experience of CMR. LV ejection fraction, mass, end-diastolic volume (EDV), end-systolic volume (ESV), and stroke volume (SV) were measured, and both volume parameters and mass were reported as indexed to the subject’s body surface area (BSA). An LV mass-to-volume ratio was calculated by dividing the LV mass by EDV. An LV global function index (LVGFI) was derived from the following formula: LVGFI = [LVSV/((LVEDV + LVESV)/2 + LV mass/1.05)]x100 [19]. An LV early diastolic peak filling rate (PFR) was obtained from the LV volume versus time curve. In diastolic dysfunction, PFR is decreased due to impaired LV relaxation and/or increased myocardial stiffness causing reduced suction effect. LV EDV normalized values of PFR (PFR/LVEDV) were also reported. The LV filling curve was visually inspected to identify the plateau between the early diastole caused by ventricular relaxation and the late diastole, the result of atrial contraction. The resulting diastolic plateau volume was divided by the EDV and the resulting percentile was reported as the ratio of early diastole. The physiological basis for measuring this index is that in the first phase of diastolic dysfunction, the proportion of LV filling in the early phase of diastole is reduced, and the contribution of left atrial contraction to LV filling is increased [20]. Diastolic dysfunction can be seen in LV volume kinetics as a depression of the diastolic plateau and as a shift from left to right in early diastole due to suppressed PFR (Figure 2).

### Quantification of myocardial TG content

For measuring the myocardial TG content, cardiac ^1^H-MRS was performed in a 1.5 T MR imager (Magnetom Avanto; Siemens AG, Erlangen, Germany) using a standard flex-coil for signal reception. The spectroscopic volume of interest was placed within the interventricular septum using the end-systolic cardiac cine images in three planes. The localizer images and spectroscopic data acquisition were double-triggered to end-exhalation and end-systole, using Prospective Acquisition Correction navigator echoes (PACE, WIP-sequence, program version B17) to control for respiratory movement and electrocardiograph-derived R wave to control for cardiac pulsation. Spectral localization and data collection were performed with the PRESS sequence with 35 ms echo time, while repetition time (TR > 3000 ms) did not fall below the respiratory cycle length. Navigator echoes were collected from the lung-diaphragm interface and the end-systole triggering was set at about 80% of the resting heart rate of the subject. The spectra were collected with and without water suppression, using 32 and 4 acquisitions, respectively, and analyzed with jMRUI v3.0 software [21] using the AMARES algorithm [22] to determine water (4.7 ppm), methylene (1.3 ppm) and methyl (0.9 ppm) resonance areas. The myocardial TG content was expressed as a ratio of fat to water (%). Correction for methylene T2 relaxation was not possible due to lack of reliable data for cardiac application.

### Quantification of epicardial and pericardial fat

**Figure 3:**
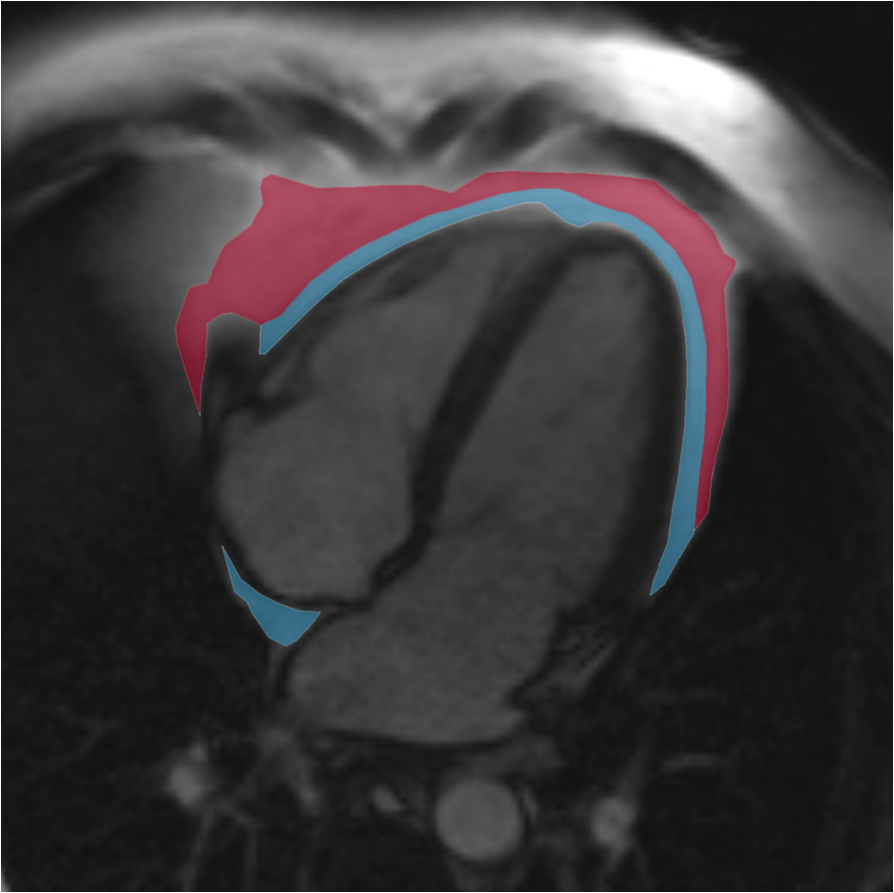
**Determination of epicardial and pericardial fat.** Contours of the epicardial (shown in blue) and pericardial (shown in red) fat were outlined in a 4-chamber oriented end-diastolic image.

The 4-chamber oriented cine images were applied for measuring the epicardial and pericardial adipose tissue area as described previously [23]. All phases of the cine images were inspected and the measurements were performed in the single end-diastolic image using a standard radiologic workstation (Impax 5.5 software, Agfa Healthcare, Mortsel, Belgium). The areas of high intensity fat layers between the myocardium and the visceral pericardium (epicardial fat) and outside the parietal pericardium (pericardial fat) were measured (Figure 3). Intra-thoracic adipose tissue outside the pericardium in the particular slice was included to the value of pericardial fat. A stack of short-axis oriented end-diastolic T1-weighted turbo spin echo images was also obtained (with typical imaging parameters of: repetition time 1050 ms, echo time 29 ms, flip angle 180 degrees, matrix 256 × 256, slice thickness 6 mm, and gap 1.5 mm), and they were additionally used to aid in epi- and pericardial fat separation as well as in water and fat separation, once needed. Intra- and inter-observer variability of epicardial and pericardial fat quantification was evaluated by two radiologists on separate occasions by the measurement of 20 (10 MetS and 10 without MetS) randomly selected study subjects.

### Statistical analyses

All statistical analyses were performed with IBM SPSS Statistics for Windows, version 19.0 (IBM Corp., Armonk, NY, USA). Normality of continuous variables was analyzed by the Kolmogorov-Smirnov test. Logarithmic transformation of variables was performed, if necessary. Data are presented as frequencies or percentages for categorical variables, as means ± SD for normally distributed continuous variables, and as medians (range) for skewed variables. Between-group differences were assessed by the Mann–Whitney *U* test, unpaired *t*-test, and the chi-square test, as appropriate. Analysis of covariance was applied to compare the means or medians of LV dimensions and function with adjustment for age. Levene’s test was used to assess homogeneity of variances. To detect determinants of myocardial TG content and epicardial and pericardial fat, univariate age-adjusted analyses were performed. The results of the correlation analyses are presented both with and without Bonferroni correction. Stepwise multivariable regression analyses were used to evaluate the impact of cardiac fat depots on LV diastolic parameters as dependent variables. In univariate analyses, epicardial fat showed a strong relationship with pericardial fat. Therefore, these variables were not forced into the same multivariate model. Differences were considered statistically significant at p < 0.05. Intra- and inter-observer variability was assessed via intra-class correlation coefficients (ICC). Absolute agreement ICCs were calculated via a two-way mixed model for single measures.

## Results

### Subject characteristics

Clinical and biochemical characteristics and measurements of cardiac fat deposits are summarized in Table 1. Participants with MetS (n = 37) were, on average, 7 years older than subjects without MetS (n = 38) and included more current smokers. Subjects with MetS had greater waist circumference and BMI, and higher HOMA index compared with the subjects without MetS. Comparison of the serum lipid profile between the groups showed higher total cholesterol, low-density lipoprotein cholesterol and TGs, and lower high-density lipoprotein cholesterol in the MetS group. In MetS, myocardial TG content was on average twice higher than in subjects without MetS. The areas of epicardial and pericardial fat were significantly larger in the MetS group in comparison with the control group.

**Table 1:** Clinical and biochemical characteristics and cardiac fat compartments in the study population

	**MetS present (n = 37)**	**MetS absent (n = 38)**	**p**
Age (years)	47 ± 6	40 ± 8	<0.001
Body mass index (kg/m^2^)	30.9 (24.2-42.5)	23.4 (17.6-29.8)	<0.001
Waist circumference (cm)	107.0 (94.0-135.0)	87.0 (71.0-93.5)	<0.001
Height (cm)	180 ± 6	180 ± 6	0.665
Current smokers (N, %)	13 (35)	4 (10)	0.014
Systolic blood pressure (mmHg)	132 ± 14	115 ± 10	<0.001
Diastolic blood pressure (mmHg)	88 ± 9	74 ± 6	<0.001
Total cholesterol (mmol/L)	5.25 ± 0.74	4.38 ± 0.80	<0.001
Low-density lipoprotein cholesterol (mmol/L)	3.25 ± 0.71	2.52 ± 0.67	<0.001
High-density lipoprotein cholesterol (mmol/L)	1.02 ± 0.26	1.50 ± 0.40	<0.001
Triglycerides (mmol/L)	2.20 (0.65-6.26)	0.72 (0.35-1.57)	<0.001
fP-glucose (mmol/L)	5.8 (4.6-6.9)	5.0 (4.4-6.0)	<0.001
fS-insulin (mU/L)	9.3 (3.3-36.9)	2.9 (0.9-7.7)	<0.001
HOMA-IR index	2.6 (0.8-8.0)	0.6 (0.2-2.0)	<0.001
Myocardial triglyceride content (%)	0.90 (0.31-2.33)	0.43 (0.14-1.39)	<0.001
Epicardial fat (mm^2^)	838 (385-1753)	518 (251-1129)	<0.001
Pericardial fat (mm^2^)	1905 (615-6131)	562 (66-1582)	<0.001

Data are expressed as means (± SD), medians (range), or frequencies (%). HOMA-IR, the homeostasis model assessment insulin resistance.

### Analysis of left ventricular function

An overview of the CMR data adjusted for age is shown in Table 2. In the MetS subjects, BSA- indexed values of LV ESV, EDV, and SV were smaller than in the subjects without MetS. LV volumes were within the normal range in both study groups, and differences remained significant after adjustment for the amount of exercise (data not shown). The LV ejection fraction was normal in all participants and comparable between the study groups. The LV mass indexed to BSA did not differ between the study groups. The LV mass-to-volume ratio was greater and the LVGFI lower in the MetS group compared to the control group, indicating concentric rather than eccentric remodeling. LV early diastolic PFR, PFR/EDV, and ratio of early diastole differed significantly between the study groups associating diastolic dysfunction with MetS.

**Table 2:** Left ventricular dimensions and function in the study population

	**MetS present (n = 37)**	**MetS absent (n = 38)**	**p**
Systolic function and dimensions			
LV ejection fraction (%)	61 ± 6	62 ± 4	0.745
LV end-systolic volume/Body surface area (mL/ m^2^)	25 ± 7	32 ± 5	<0.001
LV stroke volume/Body surface area (mL/m^2^)	40 ± 8	52 ± 7	<0.001
LV mass/Body surface area (g/m^2^)	58 ± 9	62 ± 7	0.190
LV mass/End-diastolic volume (g/mL)	0.88 (0.66-1.32)	0.73 (0.61-0.90)	<0.001
LV global functional index (%)	41 (24-51)	44 (35-54)	<0.001
Diastolic function and dimensions			
LV end-diastolic volume/Body surface area (mL/m^2^)	65 ± 13	84 ± 11	<0.001
Peak filling rate (mL/s)	471 (238-909)	667 (329-1315)	0.002
Peak filling rate/LV end-diastolic volume (s^-1^)	3.37 (1.89-5.46)	3.75 (2.63-7.23)	0.023
LV early diastole (%)	68 ± 9	78 ± 8	0.001

Data are expressed as means (± SD) or medians (range). All parameters are adjusted for age. LV, left ventricular.

### Correlation analysis of cardiac steatosis and LV diastolic function

**Figure 4:**
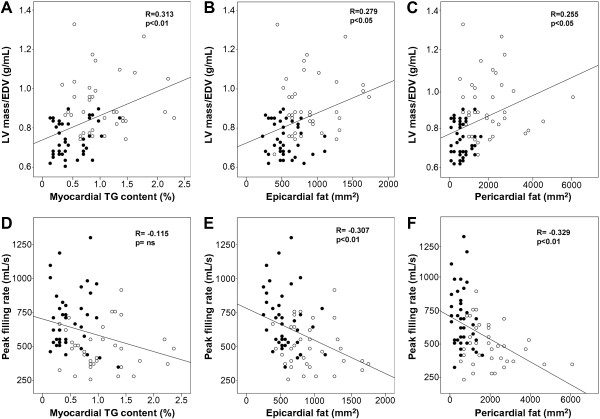
**Correlations of left ventricular mass-to-volume ratio (LV mass/EDV) and left ventricular early peak filling rate (PFR) with different cardiac fat compartments. A-C)** Relationship of LV mass/EDV and all cardiac fat compartments showing positive associations. **D)** Notice non-significant correlation of PFR and myocardial TG content, and **E-F)** significant inverse correlation between PFR and epicardial and pericardial fat. Open circles indicate subjects with MetS and closed circles subjects without MetS.

Age-adjusted univariate correlation analysis (Table 3) revealed that the amount of epicardial and pericardial fat was inversely correlated with the parameters of diastolic function. Myocardial TG content correlated with the ratio of early diastole, but not with PFR. The LV mass-to-volume ratio, end-diastolic, end-systolic, and stroke volumes indexed to BSA correlated with myocardial TG content, and with pericardial and epicardial fat. The scatter plots demonstrate that correlations of PFR with epicardial and pericardial fat are mainly due to subjects with MetS (Figure 4).

**Table 3:** Univariate correlation analysis between cardiac fat compartments and left ventricular dimensions and function adjusted for age

	**Myocardial TG content**	**Epicardial fat**	**Pericardial fat**
Fat depots			
Myocardial TG content	-	0.273^*^	0.297^*^
Epicardial fat	0.273^*^	-	0.691^‡a^
Pericardial fat	0.297^*^	0.691^‡a^	-
Systolic function and dimensions			
LV ejection fraction (%)	0.019	−0.052	0.019
LV end-systolic volume/Body surface area (mL/ m^2^)	−0.257^*^	−0.312^†^	−0.337^†a^
LV stroke volume/Body surface area (mL/m^2^)	−0.275^*^	−0.413^‡a^	−0.370^†a^
LV mass/Body surface area (g/m^2^)	−0.024	−0.235^*^	−0.225
LV mass/LV end-diastolic volume (g/mL)	0.313^†^	0.279^*^	0.255^*^
LV global functional index (%)	−0.219	−0.246^*^	−0.178
Diastolic function and dimensions			
LV end-diastolic volume/Body surface area (ml/m^2^)	−0.303^†^	−0.419^‡a^	−0.403^‡a^
Peak Filling Rate (mL/s)	−0.115	−0.307^†^	−0.329^†a^
Peak Filling Rate/LV end-diastolic volume (s^-1^)	−0.102	−0.281^*^	0.297^*^
LV early diastole (%)	−0.424^†^	−0.438^†^	−0.462^‡^

^*^p<0.05, ^†^p<0.01, ^‡^p<0.001, without Bonferroni correction.

^a^p<0.005 with Bonferroni correction.

LV, left ventricular.

Finally, we used a multivariate correlation analysis to further evaluate the interrelationship between the individual cardiac fat depots and LV diastolic dysfunction (Table 4). We found that age and epicardial and pericardial fat were all independent determinants of PFR and PFR/EDV. Age and pericardial fat were also independent predictors of the ratio of early diastole. Interestingly, once the effect of age, waist circumference, body mass index, blood pressure parameters, and epicardial and pericardial fat were taken into account, myocardial TG content was not independently related to any parameter of diastolic dysfunction.

**Table 4:** Results of stepwise multivariable regression analysis

**Independent variables**	**Model 1**		**Model 2**	
	**β**	**p**	**β**	**p**
Dependent variable: Peak filling rate (mL/s) (log)				
Age	−0.406	<0.001	−0.379	<0.001
Waist circumference (log)	−0.157	0.219	−0.164	0.214
Body mass index (log)	−0.126	0.303	−0.138	0.257
Systolic blood pressure	−0.074	0.521	−0.075	0.525
Diastolic blood pressure	−0.088	0.440	−0.089	0.442
Myocardial triglyceride content (log)	−0.024	0.830	0.002	0.989
Epicardial fat (log)	−0.318	0.002	-	-
Pericardial fat (log)	-	-	−0.316	0.003
Adjusted R^2^	**0.317**	**<0.001**	**0.310**	**<0.001**
Dependent variable: Peak filling rate/LV end-diastolic volume (log)				
Age	−0.276	0.018	−0.256	0.027
Waist circumference (log)	−0.140	0.316	−0.161	0.266
Body mass index (log)	−0.099	0.461	−0.122	0.360
Systolic blood pressure	−0.003	0.981	−0.009	0.944
Diastolic blood pressure	−0.035	0.781	−0.042	0.740
Myocardial triglyceride content (log)	−0.019	0.878	−0.002	0.989
Epicardial fat (log)	−0.303	0.007	-	-
Pericardial fat (log)	-	-	−0.287	0.013
Adjusted R^2^	**0.192**	**<0.001**	**0.178**	**<0.001**
Dependent variable: LV early diastole (%)				
Age	−0.445	<0.001	−0.422	<0.001
Waist circumference (log)	−0.375	<0.001	−0.204	0.082
Body mass index (log)	0.235	0.296	−0.107	0.328
Systolic blood pressure	0.009	0.934	−0.015	0.887
Diastolic blood pressure	−0.131	0.243	−0.154	0.132
Myocardial triglyceride content (log)	−0.087	0.442	−0.105	0.318
Epicardial fat (log)	0.015	0.897	-	-
Pericardial fat (log)	-	-	−0.405	<0.001
Adjusted R^2^	**0.432**	**<0.001**	**0.451**	**<0.001**

Epicardial fat is an independent variable in Model 1 and pericardial fat in Model 2. β = regression coefficient.

### Reproducibility of fat quantification

Intra-observer reproducibility for epicardial fat assessment was high with a ICC of 0.97 and 0.99 for pericardial fat respectively. Inter-observer reproducibility showed ICC of 0.91 for epicardial and 0.96 for pericardial fat.

In order to test the repeatability of the ^1^H-MRS with WIP-sequence, we repeated the sequence in five subjects with varying degrees of myocardial TG content. Shim values and measurement parameters were kept unchanged. The measurements correlated (R^2^ = 0.9975) with a coefficient of variation of 9.0%.

## Discussion

To the best of our knowledge, this is the first study utilizing CMR technology to combine the measurements of all three cardiac fat compartments with detailed analyses of LV function in a group of non-diabetic men free of cardiovascular disease. The main findings of the study are as follows: 1) MetS is associated with LV diastolic dysfunction; 2) MetS-linked changes in the structure of LV are rather concentric than eccentric by nature; 3) ectopic accumulation of epicardial and pericardial fat correlates with the degree of LV diastolic dysfunction, and 4) myocardial TG content is not independently associated with LV diastolic dysfunction.

In our study, MetS was strongly associated with LV diastolic dysfunction, as demonstrated by lowered LV early diastolic PFR and ratio of early diastole. In earlier studies, reduced PFR in insulin-resistant obese women and in T2DM patients have been reported [10,15]. Rider et al. reported similar findings in a female-dominant gender-mixed cohort consisting of markedly obese subjects (BMI 38.7) with unknown status of MetS [14]. Thus, the present study adds to previous knowledge on the association between MetS and LV diastolic dysfunction in non-diabetic male subjects with moderately increased BMI. Our results give further support to the role of cardiometabolic effects of visceral obesity in the development of obesity-associated LV diastolic dysfunction.

The LV mass was similar in the two study groups, but LV concentric remodeling was present only in subjects with MetS. Concentric LV remodeling has been associated with abdominal obesity and is considered as an early sign of obesity-related cardiac remodeling before the development of LV hypertrophy [24]. Furthermore, LVGFI has recently been introduced as a novel method to integrate LV structure with global function [19]. An LVGFI value of <37% was associated with a significant risk of cardiovascular events. In our study population, LVGFI was significantly lower in the MetS patients than in the subjects without MetS, also supporting the tenet that the nature of cardiac remodeling is rather concentric than eccentric in MetS.

MetS is strongly associated with an increased amount of visceral adipose tissue, which in turn is the best predictor of pericardial and epicardial fat according to our previous study [23]. An increased amount of epicardial fat is related to burden of atherosclerotic plaques, CAD (coronary artery disease) and myocardial ischemia [25,26]. This CMR study confirms that epicardial fat is also strongly associated with LV diastolic dysfunction. This is in line with earlier findings based on echocardiography and computed tomography [27]. Epicardial fat is a metabolically active fat depot in a direct contact with the coronary arteries and myocardium. Animal studies have demonstrated that it shows higher lipogenic and lipolytic activities than other fat deposits [28]. However, little is known about the physiology of lipid storage in human epicardial fat [29]. It may have a constitutive role in cardiac lipotoxicity serving to store FFAs or as a protective buffer of TG accumulation in the myocardium. Furthermore, in obese patients it secretes proinflammatory cytokines with a role in coronary atherogenesis [30]. Finally, a mechanical role for the epicardial fat may be possible to taper the myocardial relaxation or to increase the myocardial stiffness.

Most studies have focused on epicardial adipose tissue, and a lesser role has been left to pericardial or intra-thoracic fat. In our study, pericardial fat correlated with diastolic dysfunction even on a larger scale of parameters than epicardial fat. Similarly, as a marker of ectopic fat accumulation, pericardial fat has been reported to associate with insulin resistance and 10-year CAD risk more strongly than epicardial fat [31].

Interestingly, unlike other cardiac fat deposits, myocardial TG content was not independently associated with LV diastolic dysfunction in multivariate analysis where other cardiac fat deposits are taken into account. In line with our results, two studies in insulin-resistant women, both without an assessment of epi/pericardial fat, reported the link between the myocardial TG content and diastolic dysfunction as of borderline significance [15] or negative [32]. Our findings challenge the concept of myocardial lipid accumulation as an unambiguous marker of diastolic dysfunction, as suggested by earlier studies in T2DM patients where myocardial TG content was found to be associated with impaired LV diastolic function [10,11].

However, in subjects with MetS, myocardial TG content was increased up to two-fold compared with the subjects without MetS. Notably, the range of myocardial TG content (0.14-2.33%) was relatively narrow. Rather than a stable fat deposit, myocardial TG is a highly dynamic lipid pool, where up to three or four-fold increase has been reported following 48–72 h fasting in lean subjects [5,33]. On the other hand, short-term lipid excess did not increase TG content in cardiomyocytes [9]. In diabetic subjects, 16 weeks calorie restriction decreased myocardial TG levels and improved diastolic function [33]. Hence, myocardial TG content may be adaptive to both short and long-term dietary interventions, and it may thus serve as a relatively rapidly changing reservoir of energy, in an analogous fashion to intramyocytic TG of skeletal muscle [34]. Normal heart utilizes FFAs and glucose as main energy sources with a ratio of 3:1 [35]. In the setting of obesity, the energy balance is shifted even more from glycolysis toward the increased β-oxidation of fatty acids [36]. In MetS, the increased intramyocardial TG may actually reflect the enhanced demand of lipids due to the preference of using fat more over glucose as the primary fuel of the heart.

In conclusion, our study suggests that although myocardial TG is elevated in subjects with MetS, it cannot be used as a surrogate parameter for cardiac function. Further studies are needed to examine the dynamics of intramyocardial lipids, and elucidate the role of their excess in the myocardium.

### Limitations

To exclude the effects of hormonal variability, our study population was limited to men. Recently, the association of myocardial TG content and obesity has been studied also in female subjects [15,32]. In our study, subjects with MetS were older than the controls producing a potential source of bias for the evaluation of cardiac steatosis and diastolic dysfunction. However, our results remained significant after adjusting for age. There was a clear difference in biomarkers between subjects with and without MetS, however, the differences other than MetS criteria might be significant confounders in terms of differences in LV structure and function. Although the measurements of diastolic function consisted of multiple parameters, they were based solely on LV short axis cine images. Other methods such as trans-mitral velocity-encoded flow analysis might have served as an internal reference. However, pitfalls of this technique include a pseudonormal E/A-pattern related to diastolic dysfunction, and common averaging errors of velocity measurements due to data gathering time of several minutes and intra-cycle variation. The measurement of the epicardial and pericardial fat may be prone to bias as it did not cover the entire volume of the particular fat tissue. However, in our preliminary data, this method correlates well with conventional but very time-consuming Simpson method [23]. Finally, the cross-sectional nature of the study design limits inferences of causality.

## Conclusions

Metabolic syndrome associates with both structural and functional changes in the heart leading to LV diastolic dysfunction. The amount of epicardial and pericardial fat correlates with the severity of these changes. Myocardial TG increases as well, but it does not seem to act as a mere fat deposit in the same way as epicardial and pericardial fat. Instead, our study favors a more complex role for myocardial TG in obesity associated cardiovascular diseases.

## Abbreviations

BMI: Body mass index; BSA: Body surface area; CAD: Coronary artery disease; CMR: Cardiovascular magnetic resonance; EDV: End-diastolic volume; ESV: End-systolic volume; FFA: Free fatty acid; 1H-MRS: ^1^H- magnetic resonance spectroscopy; ICC: Intra-class correlation coefficient; LV: Left ventricular; LVGFI: Left ventricular global function index; HOMA: Homeostasis model assessment; MetS: Metabolic syndrome; PFR: Peak filling rate; SV: Stroke volume; T2DM: Type 2 diabetes mellitus; TG: Triglyceride.

## Competing interests

The authors declare that they have no competing interests.

## Authors’ contributions

MRT conceived the study and all other authors assisted with the study design. KN and KL were responsible of CMR data and analyses, and JL and AH carried out MRS acquisition and analyses. MG, MP, and RS were involved in the subject recruitment and screening. MG performed statistical analyses and MG, MRT, KN, MP, and KL contributed to the data interpretation. KN and MG assembled the figures and tables. KN drafted the manuscript with the help of MG, MP, and KL, NL, MRT, KL, and MN provided supervision and expert advice, and revised the manuscript critically for important intellectual content. All authors read and approved the manuscript.
